# Identifying and Overcoming Policy-Level Barriers to the Implementation of Digital Health Innovation: Qualitative Study

**DOI:** 10.2196/14994

**Published:** 2019-12-20

**Authors:** Laura Desveaux, Charlene Soobiah, R Sacha Bhatia, James Shaw

**Affiliations:** 1 Institute for Health System Solutions and Virtual Care Women's College Hospital Toronto, ON Canada; 2 Institute for Health Policy, Management, and Evaluation University of Toronto Toronto, ON Canada

**Keywords:** health care, policy, implementation, health technology

## Abstract

**Background:**

High-level policy barriers impede widespread adoption for even the most well-positioned innovations. Most of the work in this field assumes rather than analyzes the driving forces of health innovation. Objective: The aim of this study was to explore the challenges and opportunities experienced by health system stakeholders in the implementation of digital health innovation in Ontario.

**Objective:**

The aim of this study was to explore the challenges and opportunities experienced by health system stakeholders in the implementation of digital health innovation in Ontario.

**Methods:**

We completed semistructured interviews with 10 members of senior leadership across key organizations that are engaged in health care–related digital health activities. Data were analyzed using qualitative description.

**Results:**

A total of 6 key policy priorities emerged, including the need for (1) a system-level definition of innovation, (2) a clear overarching mission, and (3) clearly defined organizational roles. Operationally, there is a need to (4) standardize processes, (5) shift the emphasis to change management, and (6) align funding structures.

**Conclusions:**

These findings emphasize the critical role of the government in developing a vision and creating the foundation upon which innovation activities will be modeled.

## Introduction

### Background

Digital health innovation is the cornerstone of health care modernization efforts in a number of countries internationally [1-3]. Notwithstanding select evidence of impact on quality and efficiency [4,5], its application in practice has been described as a *plague of pilots* where innovations fail to become part of routine practice because of limited funding or inability to scale to broader sectors of the health care system [6-11]. Translating evidence into practice remains a challenge despite the accumulating body of evidence regarding factors underlying success and failure [12,13]. The Ontario government began developing the Digital Health Strategy in 2015 and unveiling the strategy and its guiding principles in 2016 (see Table 1). In this strategy, the Ontario government outlined a Digital First philosophy as 1 of the 7 guiding principles, which considers new and existing programs and asks, “How can we do it with digital health?” [14]. The creation of this strategy addressed a previously unmet need for provincial guidance on the adoption and integration of digital innovation within the public system, signaling a potential inflection point that hinges on the ability to both understand and overcome barriers to digital health innovation.

**Table 1:** Guiding principles for the Ontario digital health strategy.

Principle^a^	Description
Put patients first	Focus efforts on faster access to care, innovative and integrated care, empower patients and caregivers, and ensure a fiscally sustainable public health care system
Adopt a “Digital First” philosophy	Approach new and existing programs and discussions by asking, “How can we do it with digital health?”
Make solutions about quality care	Design new policies, care models, funding structures, and workflows that are best for patients and providers—then think about how technology can help
Be transparent	Use open, evidence-based standards to guide governance and investment decisions; report progress publicly and regularly
Be innovative	Use the full scope of creativity of what is possible with contemporary technology to support patient and provider decision making, virtualize processes, and deliver services
Build on what we have already	Leverage existing assets as a starting point when possible
Be pragmatic	Strive for solutions that are “good enough” and processes, such as governance, that are not needlessly burdensome

^a^Adapted from Bell R. Ontario’s Patients First: Digital Health Strategy. Canada Health Infoway Partnership Conference 2016 [14].

### Overarching Barriers to Digital Health Innovation

Obstacles in the digital health innovation process stem from features of the innovation itself and the broader implementation context, which includes the divergent interests of a variety of stakeholder groups [14]. Innovations that establish proof of concept often lack a strategic plan for diffusion, which complicates uptake and adoption into routine care [15,16]; however, high-level policy barriers impede widespread adoption more broadly for even the most well-positioned innovations [17]. For the purposes of this paper, we refer to adoption at the organizational level, recognizing that this cannot be achieved without engagement of and support from frontline clinicians. Context and culture drive changes in the use of technology [18], highlighting the central role that policy reform will play in the success (or failure) of the digital health agenda. Despite this, policy (or a lack thereof) is conspicuously missing from studies examining the drivers of failure when it comes to scaling digital health innovations. For example, Sundin et al [11] categorize failures according to financial, technical, organizational, employee, customer, or contextual barriers.

### Policy is a Central Driver of Change

Greenhalgh et al [19] highlight that health and fiscal policy often underpinned the inability to move from a successful pilot to mainstream service and explicitly outline the need to identify and attend to potential drivers (or roadblocks) at a policy level to avoid nonadoption or abandonment. Attention has been paid to practical guidance for health system modernization, outlining approaches to funding reform and the organization of services [20]. The same attention and rigor devoted to the mechanics of the activity must be paid to the overarching policy context [21]. An array of organizational and institutional arrangements underpins the innovation process, which includes both innovation development and facilitating the implementation of advancements into clinical practice [22]; however, most work assumes rather than analyzes the underlying components and driving forces of health innovation [22]. Guiding theories, such as Rogers Diffusion of Innovation [23], highlight that the process of adoption is at the level of the individual, which is driven by communication through social channels. Diffusion occurs across several stages that include knowledge, persuasion, decision, implementation, and confirmation, highlighting that an individual’s decision to adopt or reject an innovation depends on receiver variables, social system variables, and perceived characteristics of innovation [23]. Organizational factors, such as the capacity to innovate, readiness for the digital health innovation, availability of funding, and extent of changes required to implement the innovation [20], also influence the adoption process [24] and thereby impact the uptake of digital health innovations in practice. Therefore, the objective of this study was to understand the challenges and opportunities in the implementation of digital health innovation from the perspective of organizations as the adoption unit, and the potential policy-level actions that might promote enhanced uptake of digital health innovation at scale in Ontario.

### Shifting From an Economic to a Health System Lens

The major contributions to the body of literature on *Health Innovation Systems* are driven by an economic perspective and thereby frame innovation achievements against the backdrop of economic change [22]. In contrast to that work, we are focused on innovations that enable *health systems* to better achieve their goals of providing better outcomes alongside improved patient and provider experience, at controlled or reduced costs [25] (ie, a digital innovation that allows for real-time image sharing across institutions and providers, reducing the need for duplicate imaging). In this way, we fundamentally adopt a health system perspective in our paper, as opposed to a view that might focus on economic development more generally. For the purposes of this paper, a health system is defined as a functionally related group of interacting organizations and providers that share a common aim [26] of providing the best possible care for a population at the lowest possible costs.

## Methods

### Study Design

Our approach was informed by a constructivist paradigm [27] and used thematic analysis [28] to understand how current challenges and opportunities impact the implementation of digital health innovations in Ontario. For the purposes of this study, we focused on the organization as the “adopter” and therefore the subsequent unit of study. These insights informed subsequent recommendations to overcome these challenges and capitalize on opportunities. Ethical approval was obtained from the Women’s College Hospital Research Ethics Board.

Our study was conducted in the province of Ontario, Canada, where the majority of health care is publicly funded and privately delivered. However, despite the fact that our data collection took place in a single Canadian province, we focused our analytic strategy on identifying challenges and opportunities that apply to health care systems across high-income countries.

### Participants

A purposive sampling strategy was used to select participants, whereby the research team generated a preliminary list of key organizations that are engaged in health care–related digital health activities in Ontario, Canada. Participants were required to occupy a position of senior leadership within their organization to ensure their ability to speak to system-level barriers. This list was then circulated to a broader advisory group to elicit suggestions to ensure a wide range of perspectives. A total of 9 potential participants were then sent an introductory email, outlining the purpose of the study and requesting their participation or asking them to identify an appropriate alternative within their organization. All 9 potential participants expressed interest and contacted the study authors directly to be scheduled for an interview.

### Data Collection and Analysis

Interviews were conducted in person by 2 experienced qualitative scientists (LD and JS). A semistructured interview guide was used (see Multimedia Appendix 1). The interview questions were open ended to elicit participant experiences related to the policy and strategy dimensions of the implementation of digital health innovations. Interviews were audio recorded and transcribed verbatim. A total of 3 team members (LD, CS, and JS) analyzed data iteratively and inductively. The first 4 transcripts were independently read and coded before the meeting to discuss an initial coding scheme. As interviews continued, original themes identified in the first 4 interviews were further explored and refined to the point of theoretical saturation while giving participants an opportunity to identify new insights. No new themes emerged after the initial 4 interviews; therefore, no additional participants were recruited after initial themes reached saturation. The findings are presented in terms of the key aspects of policy, which require attention to best promote the adoption of digital health innovations at a system level.

## Results

### Participant Breakdown

A total of 10 participants were interviewed across 9 interviews (1 participant invited a colleague to their interview), with an average duration of 40 min (range 21-61 min). Participants included representatives of key organizations within the digital health landscape (see Table 2 for descriptions), including the Ontario Ministry of Health and Long-Term Care (MOHLTC), the Ontario Telemedicine Network, Ontario MD, Canada Health Infoway, and the MaRS Excellence in Clinical Innovation Technology Evaluation program, as well as key leaders in health innovation.

Participants were unanimous in their belief that “what we’re doing at a system level is not working.” The importance of strong leadership at an organizational and system level was viewed as critical for the successful implementation of digital health innovation, with an emphasis on establishing a culture of innovation. Participants described 6 key priorities requiring action at the policy level to catalyze digital health innovation, including the following: (1) a system-level definition of innovation, (2) a clear overarching mission for digital health innovation, and (3) clearly defined organizational roles. Operationally, there is a need to (4) provide guidance on standardized processes, (5) shift the emphasis to change management, and (6) align funding structures. A participant summarized the problem as follows:

It’s still not a case of build it and they will come. I’ve been working in this space for 20 years and truly if you look at the penetration of virtual care—there’s still tremendous opportunity at the system level ...you know we are a broken system.

P03

**Table 2:** Organizational representation.

Organization	Description^a^
Ontario MOHLTC^b^	Provincial ministry responsible for administering the health care system and providing services to the province of Ontario
Ontario MD	Helping physician practices advance electronic medical records, products, and services so that we collectively enhance the delivery of patient care
Ontario Telemedicine Network	Develop and support telemedicine solutions that enhance access and quality of health care in Ontario and inspire adoption by health care providers, organizations, and the public
Canada Health Infoway	Improve the health of Canadians by working with partners to accelerate the development, adoption, and effective use of digital health solutions across Canada
MaRS EXCITE^c^	Foster the adoption of innovative health technologies in Ontario and leverage those successes and experiences into global markets

^a^Descriptions reflect organizational missions taken directly from respective organizational websites where available.

^b^MOHLTC: Ministry of Health and Long-Term Care.

^c^EXCITE: Excellence in Clinical Innovation Technology Evaluation.

### A System-Level Definition of Innovation Is Needed to Align Innovation Efforts

Innovation was defined differently across participants in our sample, with each definition exhibiting unique nuances that reflected the participant’s past experience and organizational perspective. For example, one arm’s-length policy organization was focused on understanding and modifying components of the health innovation system that could better promote the generation, testing, and ultimate adoption of new technologies:

The real innovation for us is the way of aligning all of the bits and pieces of the sector—from everything from policy and payment all the way down to the actual solution.

P05

In contrast, a representative from yet another organization took an even broader approach, defining innovation simply as changing processes of problem solving:

People are now understanding that innovation is just doing things differently—right, like changing your process, changing you’re approach, changing how you think about the problem and what you do to solve that problem.

P08

These varied definitions of innovation across key stakeholders are a consequence of a nonexistent, shared conceptual foundation for both digital health innovation and what the health system is supposed to do more broadly. This lack of shared understanding about the nature of health innovation impedes effective communication and collective action, making it extremely difficult to achieve alignment across activities.

### A Clear Mission is Needed to Drive Innovation Efforts

Drivers of innovation varied across participants and were largely reflective of their organization’s current direction and leadership. Approaches to virtual care were primarily driven by the needs of these individual organizations (ie, reduced cost or improved efficiency). The importance of patient experience was highlighted by several participants, but it was rarely highlighted as the primary driver for innovation. The tension between system needs and patient benefit was accentuated by the nature of a publicly funded health care system, where the distinction between payer and end user complicated the value proposition:

It’s classic virtual care things where the benefits accrue to the patient largely but the patient doesn’t pay. So any time you’ve got that not perfect alignment in incentives, then you’ve got work to do. To try and figure out how to get people motivated to grow the service.

P05

Virtual care initiatives were characterized by a top-down approach, despite the recognition that a “grassroots” or “frontline” approach to innovation is more likely to support effective problem solving and adoption. Despite highlighting clinician resistance as a key barrier to adoption, participants often described decision-making processes that failed to engage relevant end users (ie, clinicians and/or patients):

One of the biggest groups that resists process is clinicians. The way they function—they’re workflow, is disrupted when you put in a disruptive technology—so that’s one of the difficult groups [...] so you know that is sort of one area that we would be struggling with likely in all our technologies is the end user of the technology.

P07

### Organizational Roles Need to Be Clearly Defined

Participants described a poorly organized system with respect to the introduction, adoption, and scale of virtual care innovations. The key players within the system’s virtual care space are fragmented and function strategically and operationally as independent organizations. Participants felt that unclear roles and responsibilities perpetuated this fragmentation, and they proposed effective governance and accountability as a potential solution:

I think—in Ontario—this is a real problem is because ownership is often not taken or not clear, and so who’s driving that agenda is not clear- and [who is] accountable for it and when they do become accountable for that. [Organizations] take a very narrow space of it, where it’s just their thing that they can do and that’s a problem.

P09

The MOHLTC’s Digital Health Board, an advisory committee tasked with providing advice with respect to priorities, was described as a “sponsor” of the province’s digital health strategy but devoid of “any formal accountability.” Participants emphasized that, although priority setting begins to address the issue, a general lack of accountability persists, which hinders collaboration and progress. In extreme cases, this leads to organizations having competing or overlapping priorities, resulting in an inefficient use of system resources:

There’s another layer around prioritization around the big agencies in this eHealth space, and the ministry did say these are your roles in a letter last year to all of us, that has never been enforced, we’re kind of still figuring it out.

P01

### Provide Guidance on Processes to Standardize Across Organizations

The fragmented nature of processes and infrastructure related to virtual care was attributed to the operational silos that characterize virtual care organizations and health care institutions. Fragmentation results in a virtual care landscape that includes a heterogeneous assortment of technologies with limited interoperability, driven by disparate, institutionally specific procurement processes that are widely acknowledged as onerous and not conducive to early-stage innovations:

Every different hospital is different, taking a different approach, working with different partners, and in some respects, that’s promoted by the chief innovation officer of programs is that they do want institutional partnership between institutions and innovators—but that ends up being less collaborative across institutions.

P09

In the absence of a shared vision and shared processes, organizations engage in procurement decisions independent of one another, which contributes to the lack of interoperability among technological innovations within the broader system. This was unanimously viewed as a significant barrier to a virtually enabled health care system, complicating the landscape for new innovations for which interoperability is fundamental to their functionality and value proposition.

### The Emphasis Needs to Shift From the Technology to Change Management

The existence of microcultures within organizations (and therefore the system) presents both an opportunity and a challenge, as some of these microcultures push for change, whereas others try to maintain the status quo. Strategies to enable a broader culture shift included collaborative approaches to innovation, entry-level education, and modifications to existing incentives:

Basically they are different elements of the system and different structures in the system and any time you try to make change there is a tendency for those individual structures or nodes to try to revert back to the current state—the status quo. So, there’s a kind of system stability. I think that it’s possible to give sufficient pushes at different nodes and changing the incentives at each node to move to the different state within the system.

P02

Establishing buy-in from clinicians is “*all about the change management.*” The importance of this shift in mindset from implementation to change management was recognized by many participants; however, it was only operationalized by a few. The current emphasis was primarily placed on the solution and the proposed payment model—a mindset that was viewed as a barrier to successful adoption. Although the payment model was highlighted as a key barrier to adoption and scale (as the system lacks a mechanism for clinicians to bill for virtually enabled care), this was not viewed as a significant challenge from a change perspective:

Just changing the care model—or the payment model—will not make that happen, you have to actually have an adoption plan and you know to actually promote that to occur and so there really has to be change management strategies to make that occur—so you have to have both of them to make that actually happen.

P09

### Funding Mechanisms Must Evolve to Reflect the Nature of Innovation

Siloed funding for virtual care initiatives and innovation further contributes to the fragmentation of activities across the sector. Siloed program funding creates a barrier to establishing a business case, as many virtual solutions that are designed for one setting (eg, the community) will result in savings realized in another setting (eg, acute care):

There aren’t many mechanisms in place where they can flow budget from one group to another and when you’ve got these silos around the way dollars flow, that can be a real hurdle in how innovation is taken up.

P07

The primary funding mechanisms for organizations interested in innovation are institutional operating budgets or public grant funding through national agencies (ie, Canadian Institutes of Health Research). Unfortunately, institutional budgets are considerably strained, and *“*there are very few central points of knowledge around how [grant] funding works*”* across the range of funding sources. Funding is usually given for a defined period, and it leaves the responsibility of sustainability and ongoing funding to the organization itself. Precarious funding impacts the likelihood of sustainability and results in siloed investments that ultimately undermine the implementation and adoption of innovation efforts.

## Discussion

### Advancing the Understanding of Policy-Level Barriers to Digital Innovation

Our results build on previous literature by illustrating how a lack of system guidance, both conceptually and structurally, contributes to the inability of many digital health innovations to move beyond local success to realize their impact at scale. Despite technological advances and rapidly accumulating evidence on the value of digital health, the development of policy-level guidance has lagged behind. Against the backdrop of Ontario’s *Digital First* strategy, policy-level gaps undermine the potential success of digital health innovations. First, there is a need for system-level clarity around the definition of innovation, the primary mission underlying innovation efforts, and organizational roles and responsibilities. In addition to these governing principles, the strategies to support the uptake of innovation in practice must evolve to align with the objectives of the broader system. These strategies include, but are not limited to, organizational procurement processes, funding models and innovation incentives, and broader implementation strategies. As specific recommendations for funding reform (the finding *Funding Mechanisms Must Evolve to Reflect the Nature of Innovation*) have been outlined previously [20], we will devote the discussion to exploring the remaining recommendations.

### Establishing a Definition of Digital Health Innovation

Health care organizations’ pursuit of their missions is often fraught with complexity. Failure to achieve full realization often extends beyond funding issues and is attributable to organizational structures and interactions or competing policy pressures [29,30]. An overarching definition of innovation and its agenda are needed at a system level to help organize and align innovation efforts across the many organizations that make up the system. Innovation refers to novel products, processes, business models, methods of communication, or origination of novel markets (ie, those that were not previously known or used in a given setting) [31]; therefore, digital health innovation could be described as novel *digitally enabled* products, processes, business models, methods of communication, or origination of new markets in health care. Novel innovations do not have to be new but can be borrowed from other industries and applied in different contexts or could be used in different ways. Digital health innovations should be evidence informed and show a positive impact to support spread and scale across organizations.

### Articulating a Clear Mission

Articulating a vision and establishing a clear direction are central to the ability to achieve health care transformation [32,33]. An absence of clearly defined goals undermines accountability [34] and the ability of individuals and organizations to achieve broader system goals. Furthermore, there is a need to clarify what should be done when conflict arises among the range of competing demands (eg, access, quality, cost control, and customer satisfaction) [35]. The implementation of innovation at scale depends on the co-ordination of various types of knowledge within a system or, more explicitly, the connection of various organizations and institutions [22]—a coordinated effort that relies on a common mission and understanding. We propose an explicitly stated guiding principle, whereby health system innovations *must improve at least one dimension of* the Institute for Healthcare Improvement’s “Quadruple Aim” [25] (outcomes, patient experience, provider experience, and cost), *without adversely affecting the remaining dimensions* from a system perspective (note that this may mean that while costs are increased in one area of the system, cost reductions are realized in another).

### Provide Overarching Guidance on Institutional Processes

The interactions among individuals, institutions, and organizations contribute to coherent trajectories of system change over time [22]. Analogous to the concept of technological interoperability, the processes and structures that guide innovation activities within the system must exhibit some degree of synergy. In the absence of synergistic processes, the resulting system complexity creates an inadvertent barrier to innovation [36]. Changes to existing institutional structures are crucial to the viability of a true *Health Innovation System,* which depends on effective co-ordination across a range of industries and specializations [22]. Much like the Ontario government’s *Digital First* philosophy [37], organizational processes should be developed (or revised) by asking “how can this align with similar processes or structures within the system?” We suggest that the system would benefit from policy-level guidance on key elements to include (ie, demonstrated system-level interoperability for institutional procurements), which will facilitate alignment across organizational activities and provide the foundation upon which organizational processes will be built [33].

### Shift the Emphasis to Change Management

**Figure 1:**
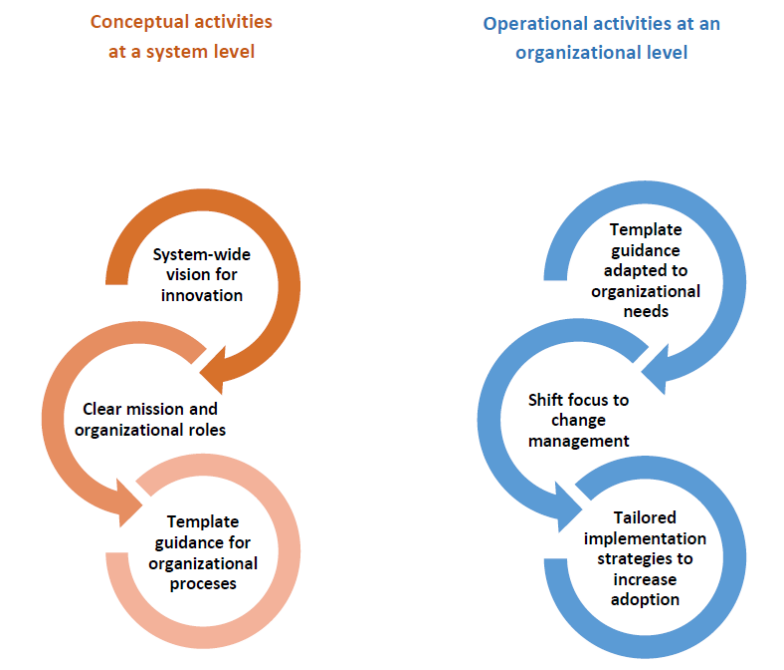
System and organizational activities to facilitate the adoption of digital health innovation.

Taking these policy-level implications of our research down to the level of the organization, we observed the following: “One-size-fits-all” strategies often translate into suboptimal engagement, underscoring the need for a change management approach that tailors implementation strategies to the varied needs of end users [38] (see Figure 1). Beyond the demonstrated need for strategies to engage clinicians and end users, a parallel need exists for investment in “internal” capabilities for transformation [39]. Implementation agents must acknowledge that the introduction of digital health innovations necessitates changes to service delivery, and change management is part of the process. As such, it is critical to attend to the central considerations of tool, team, and routine throughout the implementation process [40] to understand how successes can not only be achieved but also be spread, scaled, and sustained. The adoption of digital health innovation is an iterative process that involves complex interactions among these central factors, among others. We suggest that those individuals responsible for implementing innovations in practice utilize existing approaches [19,40-42] to assist in the systematic consideration of key factors to develop their implementation strategy in a way that mitigates the impact of unanticipated obstacles. Notably, although these tools can strengthen the development of implementation strategies, achieving transformative change through health system innovation will unequivocally require creative and bold leadership [33,36,43]. Furthermore, only 0.3% of research funding from the Canadian Institutes of Health Research (a national research funding body) has supported change management strategies or scaling up innovation [44], signaling a disconnect between system priorities and investment and underscoring the need for an aligned strategy.

It is important to note that the findings of this study depict a cross-sectional state in time. Organizations and systems are dynamic (and not time invariant); therefore, their activities are linked and informed by a grid of evolving connections. Notwithstanding, our results highlight the current system gaps, and we propose related policy-level activities that will promote the broader uptake of digital health innovation.

Although we achieved theoretical saturation in our sample, participants were mainly from urban organizations in Ontario; therefore, our findings may not reflect the challenges of implementing digital health innovations in rural organizations. Our results are not intended to be generalizable to every example of digital health innovation in Ontario, and future work would benefit from the validation or refinement of these themes from the perspectives of those responsible for technology adoption (ie, patients and health care providers). Although these findings reflect the local health system context in Ontario, Canada, many health care systems are pursuing increased quality through innovative modification of current delivery systems [2,3,20,45]. Fragmented service delivery and a lack of standardization plague health systems internationally [46], further highlighting the broad relevance of our findings.

### Conclusions

Despite much policy-level talk of triggering a revolution in service delivery and many small-scale proof-of-concept examples, digital health innovations are rarely mainstreamed or sustained [47]. Our findings build on previous work on health system capacity planning, which emphasizes the role of the government in charting the digital course by developing a vision and creating the foundation upon which (currently fragmented) innovation activities will be modeled [33]. It is important to note that transformative change does not necessitate growth in size or the addition of resources; instead, it may be achieved by thoughtful and efficient reconfiguration of existing practices [48,49]. Health care systems around the world and their stakeholders can reflect on these findings and recommendations to consider their utility in advancing local health innovation agendas. To support policy efforts, evaluations of digital health innovations should focus on identifying the factors that influence adoption of a given innovation (as outlined in Roger’s Diffusion of Innovation Theory) to support the evolution beyond the pilot stage to broader adoption.

## Appendix

Multimedia Appendix 1 Interview guide.

## Abbreviations

MOHLTC: Ministry of Health and Long-Term Care
