# Supplementary material for: Identifying and Overcoming Policy-Level Barriers to the Implementation of Digital Health Innovation: Qualitative Study
Source: J Med Internet Res. 2019 Dec 20;21(12):e14994. doi: 10.2196/14994 (PMC6942191; doi:10.2196/14994)
Supplement: Multimedia Appendix 1 [file jmir_v21i12e14994_app1.docx]

**Appendix- Study Interview Guide**

1. Tell us a bit about yourself and how your work and interests relate to virtual care.
2. Tell us about the ecosystem of organizations and individuals working on virtual care in Ontario.
3. How do you define the innovation cycle? What is involved?

- Focused questions:
  - Innovation and development
    - What do you see as the **key goal of innovation and virtual care development**?
    - What are the key considerations for people developing new virtual care technologies?
    - In your opinion, what are the current gaps in activities or thinking of those who are engaging in virtual care innovation?
      - What considerations should be top of mind for innovators?
    - What kind of policies or system issues influence what innovators focus on? (both barriers and enablers)
    - What policy level solutions could be put in place to promote innovation that most directly benefits the system?
  - Implementation, scale, and spread
    - Who is responsible for promoting the scale and spread of new virtual care technologies?
    - To what extent should scale and spread be engineered versus flow organically or naturally?
    - What kind of policies or system issues influence implementation, scale and spread? (both barriers and enablers)
    - What policy level solutions could be put in place to promote implementation, scale and spared for system benefit?
